# Cross-presentation of antigens by dendritic cells: role of autophagy

**DOI:** 10.18632/oncotarget.5268

**Published:** 2015-08-26

**Authors:** Mrinmoy Das, Srini V. Kaveri, Jagadeesh Bayry

**Affiliations:** Institut National de la Santé et de la Recherche Médicale, Paris, France

**Keywords:** Immunology and Microbiology Section, Immune response, Immunity, dendritic cells, autophagy, cross-presentation

Dendritic cells (DCs) are a heterogeneous population of antigen presenting cells(APCs) that initiate and orient immune responses. They sense the pathogens and pathogen-derived antigenic molecules via pattern recognition receptors(PRRs). They process and present extracellular antigens in the context of major histocompatibility complex (MHC) class II molecules to CD4^+^ T helper cells and intracellular antigens via MHC class I molecules to CD8^+^ T cells. DCs can also process and present extracellular antigens on MHC class I via the process of cross-presentation. Two main intracellular pathways for cross-presentation have been reported: cytosolic and vacuolar [1].

Macroautophagy, hereafter referred to as autophagy, is an important biological process involving lysosomal degradation of damaged cellular components and misfolded proteins. In this process, targeted cytoplasmic molecules will be segregated from the rest of the cellular components within autophagosome, a double-membraned vesicle structure. A series of autophagy-related proteins (ATG) and several kinases play an important role in the regulation of autophagy. Autophagy was initially investigated as a process mainly implicated in the cell survival and in the maintenance cellular homeostasis. It can also directly intersect with, and impact, major pathways of various other cellular processes including immune recognition and response. More specifically, autophagy has been identified as a route of delivering cytoplasmic and nuclear antigens to MHC class II for their presentation to the CD4^+^ T cells [2].

Role of autophagy in cross-presentation of antigens by DCs is controversial. Several reports have suggested that cross-presentation is an autophagy-dependent process while others proposed it to be autophagy-independent. These discrepancies might be attributed to various factors: experimental set-up and particularly exploring the autophagy either in DCs as such or in antigen donor cells; heterogeneity in DC subsets; and the nature of antigens used in the experiments.

The initial report demonstrated the importance of autophagy in antigen donor cells for the MHC class I cross-presentation of antigens by DCs both *in vitro* and *in vivo* [3]. The authors showed that induction of autophagy in ovalbumin (OVA)-expressing HEK 293T cells or gp100-expressing melanoma cell lines lead to enhanced cross-presentation of model antigens by murine splenic DCs to antigen-specific CD8^+^ T cells. Whereas, inhibition of autophagy in antigen donor cells greatly hampered the cross-presentation [3]. Notably, this report suggested that autophagosomes could efficiently act as antigen cargo for cross-presentation by professional APCs.

The role of autophagy in DCs towards antigen-cross-presentation met with debates. Data from CD11c^+^ conventional murine DCs where Atg5, a key gene implicated in autophagy was conditional deleted, shown that autophagy is dispensable for the innate recognition, endocytosis, phagocytosis and migration of DCs. Confirming the previous reports, autophagy was essential for the MHC class II presentation of TLR agonists-containing phagocytosed antigens. However, no defect was observed in Atg5^−/−^ DCs to cross present antigens (OVA) to MHC class I [4]. Contrary to above report, α-alumina (Al_2_O_3_) nanoparticles induced efficient cross-presentation of conjugated antigens in bone marrow-derived DCs in an autophagy-dependent manner [5].

Several murine and human DC subsets differ in their phenotype, ontogenesis and functional properties. Not all DCs but, only particular DC subsets, can cross-present antigens efficiently. Mouse CD8^+^ and CD103^+^ DC subsets are the dominant cross-presenters compared to CD8^−^ and CD103^−^ subsets. On the other hand, CD1a^+^ DCs are dominant cross-presenting subtypes than CD14^+^ in human [6]. It is thus expected that contribution of autophagy in cross-presentation is determined by subtypes of DCs that are capable of cross presenting or not.

The aforementioned pitfalls are recently addressed in an elegant way. Villadangos and colleagues report that autophagy has a role in antigen cross-presentation only in particular DC subtypes that are specialized in cross-presentation [7]. They showed that human CD1a^+^ subtype and mouse CD8^+^ and CD103^+^ DC subtypes following maturation have higher autophagy compared to CD1a^−^, CD8^−^ and CD103^−^ subtypes respectively. The autophagic flux with the accumulation of DC aggresome-like structures that contain ubiquitinated cargo and sequestosome 1/p62, one of the substrate anchors for autophagy are also enhanced in cross-presenting DCs. The authors generated *Atg* 7^−/−^ conditional knockout in CD11c^+^ DCs to assess the role of autophagy in cross-presentation by CD8^+^ DCs. The absence of autophagy did not impact the expression of MHC I, MHC II and CD86 in CD8^+^ DCs, suggesting that autophagy is dispensable for DC homeostasis in steady state. Interestingly, they found that the role of autophagy in efficient cross-presentation depends on the type of antigen. Only soluble OVA cross-presentation was impaired by autophagy deficiency, while cross-presentation of cell-associated antigens and those delivered via endocytic receptors (such as DEC-205) was unaffected. These data thus suggest that autophagy contributes to efficient cross-presentation of soluble antigens that are internalized by micropinocytosis or pinocytosis, but not by phagocytosis and receptor-mediated endocytosis.

In summary, role of autophagy in antigen cross-presentation by DCs is determined by particular subset of DCs and nature of the antigens.
